# Development of eugenol derivatives with 5-LOX inhibitory activity

**DOI:** 10.1080/14756366.2025.2535586

**Published:** 2025-08-21

**Authors:** José L. Pereira Filho, Renato B. Pereira, Tatiana F. Vieira, Sérgio F. Sousa, José R. A. Coelho, Nuno F. S. Pinto, Catarina M. M. Coelho, Maria José G. Fernandes, Elisabete M. S. Castanheira, Maria S. T. Gonçalves, David M. Pereira

**Affiliations:** ^a^ REQUIMTE/LAQV, Laboratório de Farmacognosia, Departamento de Química, Faculdade de Farmácia, Universidade do Porto, Rua de Jorge Viterbo Ferreira, Porto, Portugal; ^b^ LAQV/REQUIMTE, BioSIM – Department of Medicine, Faculty of Medicine, University of Porto, Alameda Prof. Hernâni Monteiro, Porto, Portugal; ^c^ Centre of Chemistry Department of Chemistry, University of Minho, Braga, Portugal; ^d^ Centre of Physics of Minho and Porto Universities, University of Minho, Braga, Portugal

**Keywords:** Eugenol, 5-lipoxygenase, *in silico*, chemometrics

## Abstract

Eugenol (4-allyl-2-methoxyphenol), is the major chemical constituent in the essential oil of numerous plant species. Several biological properties have been described for this molecule, including modulation of enzymatic targets relevant for the inflammatory response, such as 5-lipoxygenase (5-LOX). As so, there is interest in expanding the chemical space of this molecule to develop new molecules to be used in inflammatory conditions. We describe the chemometric analysis of several eugenol derivatives, which show that the chemical space of the parent molecule was successfully expanded. All molecules were evaluated for their inhibition towards 5-LOX, an important player in inflammatory pathways. Four derivatives exhibited significant 5-LOX inhibitory activity, which prompted further studies. The most promising compounds, 4-allylbenzene-1,2-diol **2**, ethyl-4-(4-allyl-2-methoxyphenoxy)butanoate **4e**, 3-(2-methoxy-4-(oxiran-2-ylmethyl)phenoxy)propyl acetate **5d** and 4-(3-(*tert*-butoxy)-2-hydroxypropyl)-2-methoxyphenol **7c**, were submitted to *in silico* assays to validate their affinity and stability towards 5-LOX, which helped clarify the mechanism by which these molecules interact and inhibit this enzyme.

## Introduction

Inflammation is a dynamic, multifactorial physiological process that arises in response to an infection or injury to the body in an attempt to eliminate or limit the spread of the injurious material or agent and promote tissue repair^1^^,^^2^. Under physiological conditions, the inflammatory cascade is a protective attempt by the body to start the healing process and remove harmful *stimuli*. However, if not addressed properly, inflammation can result in persistent tissue damage from infiltration of leukocytes, lymphocytes, or damaged collagen^3^.

Nonsteroidal anti-inflammatory drugs (NSAIDs) are commonly used to treat pain and inflammation^4^. Pharmacological effects of these molecules include suppression of prostaglandin biosynthesis from arachidonic acid through inhibition of cyclooxygenases (COXs), and inhibition of the biotransformation of arachidonic acid to leukotrienes (LTs) via 5-lipoxygenase (5-LOX). In this way, these drugs are capable of inhibiting key enzymes thus resulting in anti-inflammatory and analgesic effects^4^. So far, the only 5-LOX inhibitor approved for clinical use is zileuton, but its use is limited due to low pharmacokinetics and hepatotoxicity^5^. As long-term therapy with NSAIDs can cause gastrointestinal (GI) complications and some liver or kidney dysfunction, it is important to keep the search for new anti-inflammatory (AI) agents with potential clinical use and with lower incidence of adverse effects.

Medicinal and aromatic plants (MAPs) constitute a large part of the natural flora and are considered an important resource across several fields, such as the pharmaceutical, aroma and fragrance, perfumery, and cosmetics industries^6^. These plants have a chemical composition rich in secondary metabolites that play an important role in plant physiology and are targets of studies due to their biological properties, making them a promising source of new molecules of therapeutic interest^7^.

Essential oils (EOs) obtained from MAPs are aromatic in nature due to a mixture of several chemical substances that belong to different chemical classes, including aldehydes, alcohols, esters, phenolics, ethers, amines, amides, ketones and mainly terpenes^6^^,^^8^. EOs also contain non-terpene compounds that are biosynthesized via the phenylpropanoid pathway, such as eugenol, cinnamaldehyde and safrole^8^. EOs are recognised for their diverse biological activities, such as antimicrobial, analgesic, sedative and anti-inflammatory^9^^,^^10^.

Eugenol (4-allyl-2-methoxyphenol) is a phenylpropanoid and the major chemical constituent present in the essential oil of *Syzygium aromaticum* (L.) Merr. & L. M. Perry., popularly known as “clove” ^11^. Eugenol has been used in numerous applications in the pharmaceutical, food, agricultural and cosmetic industries due to its multiple biological properties, such as antimicrobial^12^, antioxidant, anti-inflammatory^11^, and antiviral^13^. This wide spectrum of biological activities makes eugenol a target molecule for structural modifications in order to produce substances with more potent therapeutic properties^12^.

Considering all the above facts, in this present work, several semi-synthetic derivatives of eugenol – namely, esters from various (hetero)aromatic carboxylic acids, *O*-alkylated carrying the propyl chain with hydrogen, chlorine, hydroxyl, ester and carboxylic acid as terminals, as well as the corresponding *O*-alkylated oxiranes, β-alkoxy alcohols, β-amino alcohols, among other derivatives – were synthesised with the aim of potentiating the anti-inflammatory capacity of the original molecule by the means of 5-LOX inhibition. Therefore, the *in vitro* biological activity of all compounds was tested against the enzymatic activity of 5-LOX, also benchmarking them with the structure of the parent molecule. Moreover, computational studies were performed to identify the most likely protein interaction with 5-LOX by applying a structure-based virtual screening protocol combined with molecular dynamics simulations and free energy calculations.

## Materials and methods

### Standards and reagents

TLC analyses were carried out on 0.25 mm thick precoated silica plates (Merck Fertigplatten Kieselgel 60F254, Germany), and spots were visualised under UV light. Chromatography on silica gel was carried out on Merck Kieselgel (230-240 mesh). Deuterated solvents were performed by Eurisotop (Cambridge, England). Disodium phosphate, ethanol, methanol, petroleum ether, dichloromethane, dimethyl sulfoxide (DMSO), quercetin, linoleic acid, soybean lipoxygenase (*Glycine max* L Merr. (V-S type; EC 1.13.11.12), L6632-1MU), and reagents used in the synthesis were obtained from Sigma-Aldrich (St. Louis, MO, USA), Fisher Scientific (Geel, Belgium) and PanReac Applichem (Barcelona, Spain). Stock solutions of all compounds under study were prepared in DMSO at a concentration of 80 mM.

### Analytical instruments

The NMR spectra were obtained on a Bruker Avance III at an operating frequency of 400 MHz for ^1^H NMR and 100.6 MHz for ^13^C NMR using the solvent peak as internal reference at 25^ᵒ^C. All chemical shifts are given in ppm using *δ* Me_4_Si = 0 ppm as reference and *J* values are given in hertz. Assignments were made by comparison of chemical shifts, peak multiplicities and *J* values and were supported by spin decoupling-double resonance and bidimensional heteronuclear correlation techniques. The IR spectra were obtained on a Spectrum Two FTIR spectrophotometer PerkinElmer (USA). Mass spectrometry analyses were performed at the “C.A.C.T.I. - Unidad de Espectrometria de Masas”, at University of Vigo, Spain. All melting points were measured on a Stuart Analogue Melting Point Apparatus SMP11 (Cheshire, England).

### Synthesis of eugenol derivatives 2-9

**Scheme 1.**
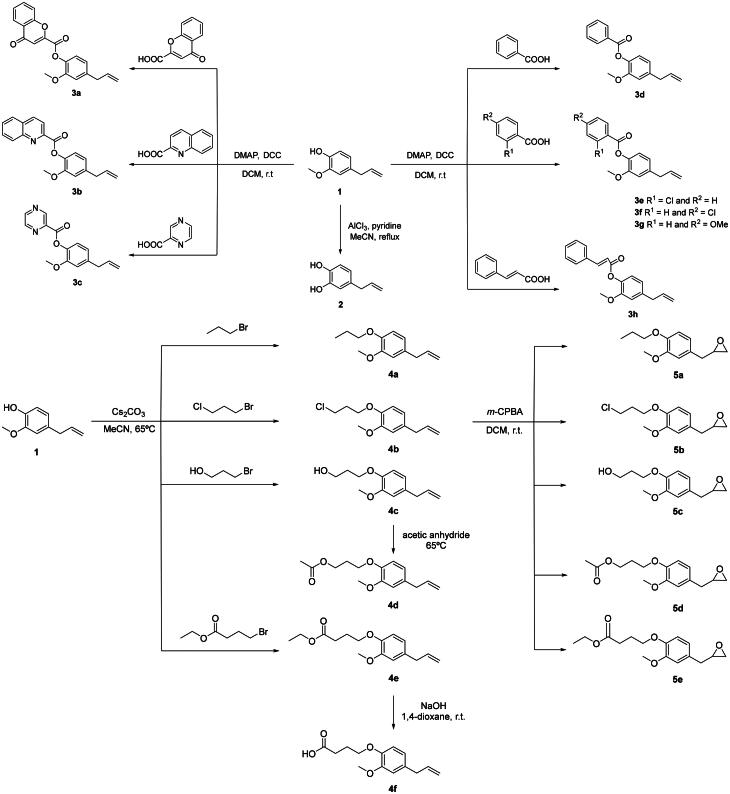
Synthesis of compounds **2**–**5**.

Compound **2** was obtained by demethylation of eugenol **1** in the presence of pyridine with aluminium trichloride using acetonitrile as solvent, by refluxing for 18h (Scheme 1)^14^.

Eugenol esters **3a**-**g** were synthesised by reaction of eugenol **1** with the corresponding carboxylic acid in dichloromethane, in presence of 4-dimethylaminopyridine (DMAP) and *N*,*N*′-dicyclohexylcarbodiimide (DCC), at room temperature, during 24 h^12^.

Compounds **4a**-**c** and **4e** resulted from the *O*-alkylation of eugenol **1**, with 1-bromopropane, 1-bromo-3-chloropropane, 3-bromopropan-1-ol and ethyl 4-bromobutanoate, with caesium carbonate, by heating at 65 °C, in acetonitrile. Compound **4c** was further reacted with acetic anhydride by heating at 65 °C to give derivative **4d**. Moreover, compound **4e** was hydrolysed with aqueous 1 M sodium hydroxide in 1,4-dioxane, at room temperature, to afford **4f**^15^.

The reaction of compounds **4a**-**e** with *m*-chloroperbenzoic acid (*m*-CPBA) in dichloromethane at room temperature gave the corresponding oxiranes **5a**-**e**^15^.

**Scheme 2.**
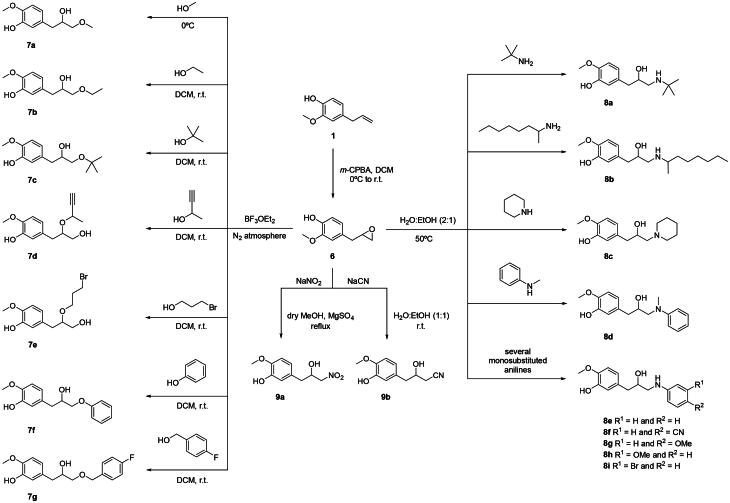
Synthesis of compounds **6**–**9**.

Starting from eugenol **1** and following the same conditions above mentioned eugenol oxirane **6** was obtained (Scheme 2) ^16^.This oxirane was further reacted with a series of aliphatic and aromatic alcohols, in the presence of a Lewis acid, under nitrogen atmosphere, at low temperature or room temperature, using boron trifluoride diethyl etherate to give the corresponding eugenol alkoxy alcohols **7a-g**^17^.The set of β-amino alcohol derivatives **8a**-**i**, were synthesised by reaction of oxirane **6**, but with a series of aliphatic and aromatic amine nucleophiles in water/ethanol (2:1) as solvent, at 50 °C^16^.

The eugenol derivatives **9a**,**b** were also obtained by reaction of oxirane **6** with sodium nitrite, under reflux, or with sodium cyanide, at room temperature, respectively^18^.

Among the compounds prepared, **3a** and **9b** are unpublished in literature and as a result, detailed experimental procedures and full characterisation of these compounds is presented.

All compounds synthesised and used in the present studies had a purity higher than 95% according to the ^1^H NMR spectra.

### Synthesis of 4-allyl-2-methoxyphenyl 4-oxo-4H-chromene-2-carboxylate 3a

A mixture of 4-allyl-2-methoxyphenol (trivialy designated as eugenol) **1** (0.500 g, 3.05 mmol), 4-dimethylaminopyridine (DMAP) (0.235 g, 1.92 mmol) and *N*,*N′*-dicyclohexylcarbodiimide (DCC) (0.944 g, 4.58 mmol) was added to 4-oxo-4*H*-chromene-2-carboxylic acid (0.871 g, 4.58 mmol) in dichloromethane (7.5 mL). The reaction mixture was stirred at room temperature for 24 h and monitored by TLC (silica: dichloromethane). At the end of this period, the white suspension obtained was filtered and the liquid phases were washed successively with 1 M hydrochloric acid (2 × 20 mL), saturated sodium hydrogen carbonate solution (2 × 20 mL) and water (2 × 20 mL). Finally, after drying with anhydrous sodium sulphate, the organic phases were evaporated under reduced pressure to give compound **3a** as a light-yellow solid (0.527 g; 51%). R_f_ = 0.64 (silica: dichloromethane), m.p. = 77–79 °C. FTIR (solid): *υ*_max_ 3070, 2934, 2857, 1758 (C = O), 1720, 1657, 1603, 1508, 1466, 1451, 1387, 1298, 1252, 1221, 1191, 1151, 1120, 1032, 944, 873, 783 cm^−1^.^1^H NMR (CDCl_3_, 400 MHz): δ_H_ 3.42 (2H, d, *J* = 6.8 Hz, C*H*_2_Ph), 3.84 (3H, s, OC*H*_3_), 5.11-5.17 (2H, m, CH = C*H*_2_), 5.96-6.02 (1H, m, C*H*=CH_2_), 6.83-6.87 (2H, m, H-3 and H-5), 7.33 (1H, s, H-3 chromene), 7.49 (1H, dt, *J* = 7.2 and 1.6 Hz, H-6 chromene), 7.66 (1H, dd, *J* = 7.6 and 1.6 Hz, H-8 chromene), 7.76 (1H, dt, *J* = 7.2 and 2.0 Hz, H-7 chromene), 8.24 (1H, dd, *J* = 7.6 and 1.6 Hz, H-5 chromene) ppm.^13^C NMR (CDCl_3_, 100 MHz): *δ*_C_ 40.08 (*C*H_2_Ph), 55.86 (OCH_3_), 112.91 (C-3), 115.82 C-3 chromene), 116.40 (CH=*C*H_2_), 118.91 (C-8 chromene), 120.79 (C-5), 122.02 (C-6), 124.53 (C-4a chromene), 125.78 (C-5 chromene), 125.98 (C-6 chromene), 134.79 (C-7 chromene), 136.79 (*C*H = CH_2_), 137.18 (C-1), 140.06 (C-4), 150.52 (C-2), 151.60 (C8a), 156.07 (C-2 chromene), 158.76 (C = O), 178.36 (C = O chromene) ppm. HRMS (ESI) calcd for C_20_H_17_O_5_ [M + H]^+^ 337.1070, found 337.1072.

### Synthesis of 3-hydroxy-4–(4-hydroxy-3-methoxyphenyl)butanenitrile 9b

To a stirred suspension of 2-methoxy-4-(oxiran-2-ylmethyl)phenol (eugenol epoxide) **6** (0.150 g, 0.83 mmol) in water/ethanol (1:1) (10 mL) sodium cyanide (0.082 g, 1.16 mmol) was added. The mixture was stirred at room temperature for 4 days, and water (10 mL) was added. The reaction mixture was extracted with ethyl acetate (2 × 10 mL), the combined organic phase was washed with brine, dried over anhydrous magnesium sulphate, and concentrated under reduced pressure to give compound **9b** as a brown oil (0.053 g; 31%). ^1^H NMR (CDCl_3_, 400 MHz): *δ*_H_ 2.46-2.60 (2H, m, C*H*_2_CN), 2.78-2.89 (2H, m, C*H*_2_Ph), 3.89 (3H, s, OC*H*_3_), 4.09-4.15 (1H, m, CH_2_C*H*(OH)), 5.67 (1H, broad s, OH), 6.70 (1H, d, *J* = 2.0 Hz, H-2), 6.72 (1H, dd, *J* = 8.0 and 2.0 Hz, H-6), 6.87 (1H, d, *J* = 8.0 Hz, H-5) ppm. ^13^C NMR (CDCl_3_, 100 MHz): *δ*_C_ 24.96 (*C*H_2_CN), 42.38 (*C*H_2_Ph), 55.91 (O*C*H_3_), 68.59 (CH_2_*C*H(OH), 111.75 (C-6), 114.70 (C-5), 117.50 (*C*N), 121.93 (C-2), 127.97 (C-1), 144.75 (C-4), 146.74 (C-3) ppm. HRMS (ESI) calcd for C_11_H_14_NO_3_ [M + H]^+^ 208.0974, found 208.0971.

### Chemometric analysis

We compiled a database of canonical smiles for all molecules. For each molecule a set of molecular descriptors were calculated using RDKit 2022.03.5^19^ and chemical structures were produced using SMILES as inputs.

In the specific case of similarity, ECFP4 fragments were generated using smiles as input. These fragments were then used to calculate Tanimoto coefficient pairwise between eugenol and each of the molecules in the library, as follows:

$${\text{SIM}}_{\text{AB}}=\frac{c}{a+b-c}$$

in which ***c*** bits set in common in the two fingerprints and ***a*** and ***b*** are bits set in the fingerprints for molecules **A** and **B.**

### *In vitro* assays of 5-LOX inhibition activity

Soybean lipoxygenase (linoleate 13S-lipoxygenase) is widely accepted as a model enzyme to screen compounds for LOX inhibition due to the high degree of binding site similarity between plant LOXs and animal 5-LOX^20^. LOX inhibition was assessed at room temperature, following the oxidation of linoleic acid to 13-hydroperoxylinoleic acid at 234 nm, according to a described procedure^21^. The assay was performed by adding 20 μL of each eugenol derivative (1.3 mM) dissolved in phosphate buffer, 200 μL of phosphate buffer (pH 9.0) and 20 μL of soybean lipoxygenase solution (100 U). After 5 min of incubation at room temperature, the reaction was initiated by the addition of 20 μL of linoleic acid (4.18 mM in ethanol), absorbance being monitored during 3 min, using a Multiskan GO plate reader (Thermo Fisher Scientific; Waltham, MA, USA). The inhibition of enzyme activity was then calculated by comparing the reaction rate with the control (no compound). Quercetin (50 μM, final concentration) was used as a positive control.

### Virtual screening

#### Structure selection and preparation

Currently there are 8 human 5-LOX crystallographic structures available in the Protein Data Bank (Table 1)^25^. At the time of the preparation of this work, 7TTJ and 7TTL were not available yet and, therefore were not considered. Two structures are bound to natural product inhibitors (nordihydroguaiaretic acid and 3-acetyl-11-keto-beta-boswellic acid) and one to the natural inhibitor (arachidonic acid). Only three of the structures do not have mutated residues. For this project, structures with no mutations and with ligands in the binding pocket were prioritised, as these are critical aspects for the success of a protein-ligand docking study^26^.

**Table 1.** X-ray structures available in the Protein Data Bank.

PDB	Mutation	Resolution (Å)	Year	Ligand	Ref.
6N2W	No	2.71	2018	Natural product inhibitors	^22^
6NCF	Yes	2.87	2018	Natural product inhibitors	^22^
3V99	Yes	2.25	2011	Natural inducer	^23^
3V98	Yes	2.07	2011	No	^23^
3V92	Yes	2.74	2011	No	^23^
3O8Y	Yes	2.39	2010	No	^23^
7TTJ	No	2.10	2022	No	^24^
7TTL	No	2.43	2022	No	^24^

#### Molecular docking

The docking program used was GOLD^27^ (with its four scoring functions (SFs): ChemPLP, ASP, ChemScore, and GoldScore). The protocol was optimised for PDB structure 6N2W. To ensure consistency, the docking conditions were the same across all the SFs tested. Re-docking was employed as a validation tool, to analyse whether the SFs can reproduce the crystallographic ligand pose’s geometry and orientation. An evaluation of binding affinity and Root mean square deviation (RMSD) was conducted to determine the accuracy and quality of the method. A RMSD bellow 2 Å was indicative of a good docking protocol and the optimum conditions were then applied to the virtual screening protocol.

The protocol used in this study is reliable and has already been described in literature for other systems^28^.

#### Virtual screening

Eight approved drugs known to inhibit 5-LOX, retrieved from the CHEMBL database^29^ were used as reference. A list of 3768 compounds with activity for 5-LOX, also retrieved from CHEMBL database, was also considered. To narrow down and create an even test set, only compounds that met the following criteria (IC_50_ value between 0.5 and 10 nM and a molecular weight below 700 g/mol) were selected. In the end a list of 34 experimentally confirmed active molecules against 5-LOX was further analysed and the docking scores were compared to the ones obtained for the inhibitor and query sets.

The four eugenol derivatives (**2, 4e**, **5d** and **7c**) were added to the pool and all the molecules were prepared for docking using Datawarrior^30^ and OpenBabel^31^.

Using the validated docking protocol, all the 40 compounds were screened against the 5-LOX PDB structure 6N2W.The choice of this structure was based in the fact that it contains no mutations and comprises 5-LOX complexed with its natural inhibitor, providing a robust starting point for the docking validation.

#### Molecular dynamics simulations

On the four protein-ligand complexes, molecular dynamics simulations were performed using the Amber21 software. Because the structure of the protein has two gaps, an homology model was generated using the SWISS-MODEL^32^ server. A total of 143 templates were found to match the target sequence and the top 24 were used to build the model (with a Global Model Quality Estimate – GMQE - of 0.95).

The starting point of each of the MD simulations was the pose predicted in the virtual screening stage, with the GOLD/ChemPLP scoring function. Using the Leap module of AMBER^33^, the protein-ligand complexes were prepared for simulation. The test molecules (**2**, **4e**, **5d** and **7c**) were parameterised with ANTECHAMBER, with RESP HF/6-31G(d) charges calculated with Gaussian16 and the General Amber Force Field (GAFF)^34^. The protein was described with the ff14SB force field. The systems were placed in TIP3P water boxes with a minimum distance of 12 Å between the protein-surface and the side of the box. The charge of each system was neutralised with the addition of Na^+^ ions.

Prior to the production run, four minimisation stages and an equilibration stage were carried out with the systems to remove clashes.

The minimisation procedure was applied to the following set of atoms: 1-water molecules (2500 steps); 2-hydrogens atoms (2500 steps); 3-side chains of all the amino acid residues (2500 steps); 4-full system (10.000 steps). Subsequently, the systems were equilibrated into two stages: 1-NVT ensemble, the systems were gradually heated to 298 K using a Langevin thermostat at constant volume (50 ps); 2- the density of the systems was further equilibrated at 298 K (subsequent 50 ps). The production run was performed during 100 ns. It was executed using periodic boundary conditions and a NPT ensemble at constant temperature (298 K, Langevin thermostat) and pressure (1 bar, Berendsen barostat). To constrain all covalent bonds involving hydrogen atoms, an integration time of 2.0 fs with the SHAKE algorithm. The nonbonded interactions were cut-off at 10-Å throughout the entire molecular simulation procedure. The RMSD final trajectories were analysed with VMD and RMSD of the system was calculated to confirm the stability of the systems. The last 70 ns of the simulation were considered for hydrogen bonding analysis, and cluster analysis of the conformations generated.

The Molecular Mechanics Poisson-Boltzmann Surface Area (MMPBSA)^35^ was applied to estimate the test compounds free energies when bound to 5-LOX, using the MMPBSA.py script^36^. A salt concentration of 0.100 mol dm^-3^ was considered as well as an external and internal dielectric constant of 80.0 and 1.0 respectively. In addition, the energy decomposition method was employed to estimate the contribution of all the amino acid residues for each of these binding free energies. From each MD trajectory, a total of 1400 conformations taken from the last 70 ns of simulation were considered for the MM-GBSA calculations.

#### Statistical analysis

Data from all analyzes were evaluated using GraphPad Prism 8.4.3 software (San Diego, CA, USA). The statistical significance between control and treatment with the tested compounds (natural product and derivatives) was analysed by the *t*-student test. *P* values < 0.05 were considered statistically significant. Pearson correlation coefficients were calculated using SciPy v 1.9.3.

## Results

### Chemical diversity of eugenol derivatives

Following the interest in finding new anti-inflammatory agents, the present work describes a strategy that consists of structural changes of eugenol in an attempt to obtain semi-synthetic alternatives with better 5-LOX inhibitory activity. Eugenol **1** was used as a starting molecule, from which 39 additional molecules were obtained, sorted between groups **1**-**9** (Schemes 1 and 2), depending on their fundamental core.

In order to assess the degree of conservation of the eugenol molecule in the derivatives, we calculated the Tanimoto coefficient (T) for all molecules, which provides information regarding the degree of similarity between two molecules, with the value of 1 corresponding to two equal molecules. To this end, we first calculated 2D fingerprints consisting of binary vectors encoding chemical substructures, specifically Morgan Fingerprints (ECFP4), having canonical smiles as input. This information was then used to calculate Tanimoto coefficient for each molecule having eugenol as reference molecule, as highlighted in the Methods section. We then sorted the results in decreasing order.

**Figure 1.**
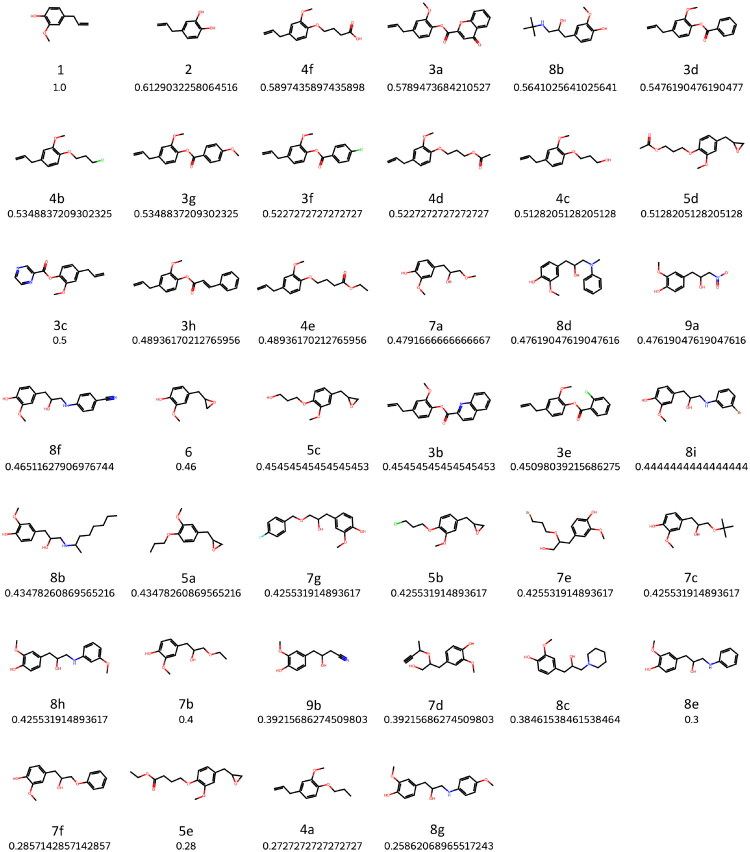
Tanimoto coefficients for all molecules in the dataset, having eugenol (**1**) as reference molecule.

Figure 1 exhibits the Tanimoto coefficients calculated, in which **2**, a catechol, is the molecule closer to eugenol (T = 0.61), a methoxyphenol.

From a chemometric point of view there is a clear distinction between the two groups in terms of physico-chemical properties. Following a chemical taxonomy approach, the majority of the molecules in the library are benzenoids (33 structures, 82.5%), followed by molecules that resemble the naturally-occurring phenylpropanoids and polyketides (5 structures, 12.5%), as classified using ClassyFire, a purely structure-based classifier^37^.

**Figure 2.**
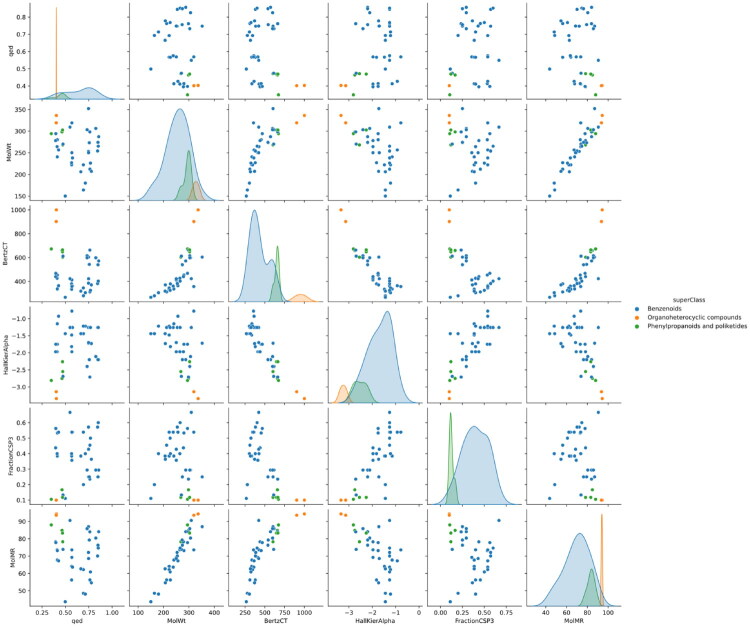
Pairwise comparison of physico-chemical and topological features of the molecules synthesised. Blue: benzenoids; green: phenylpropanoids and polyketides-like; orange: organoheterocyclic compounds. **BertzCT**: topological index meant to quantify complexity of molecules; **FractionCSP3**: fraction of carbons that are sp^3^ hybridised; **HallKierAlpha**: Hall-Kier alpha value; **MolMR**:Wildman-Crippen MR value. **MolWt**: molecular weight; **qed**: quantitative estimation of drug-likeness.

Subsequently, several physico-chemical and topographic properties of the library were calculated and computed pairwise, results being presented in Figure 2.

The results show that for several pairs of properties, there are clear differences between molecules classified as benzenoids (**1**, **2**, **4a**-**9b**), phenylpronaoids and polyketides-like (**3d**-**h**) and organoheterocyclic compounds (**3a**,**b**).

### Several eugenol derivatives are 5-LOX inhibitors

**Figure 3.**
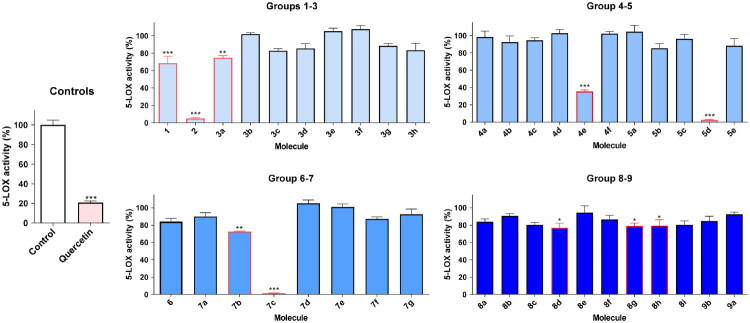
5-LOX inhibitory activity of eugenol (**1**) and its derivatives **2**-**9** (100 µM). Quercetin (50 µM) was used as a positive control for 5-LOX inhibition. * *p* < 0.05; ** *p* < 0.01; *** *p* < 0.001.

Considering the high number of molecules in the library, we conducted a preliminary screening to select compounds that presented a statistically significant potential for 5-LOX inhibition, using quercetin as a positive control (Figure 3).

After the chemometric analysis, we evaluated the ability of the molecules under study to inhibit 5-LOX in a cell-free *in vitro* assay. Out of the 40 molecules under study, 10 were shown to exhibit a statistically significant impact upon 5-LOX activity. It was observed that at a concentration of 100 μM, **7c** (Figure 3) showed almost full inhibition of the enzymatic activity of 5-LOX. **7b** scored ca. 30% of inhibition, while **7a** was inactive, the only difference among the two being the size of the chain by one carbon.

Compound **5d** was highly active, while **3f** was inactive, the only difference being the presence of an oxyran substituent group in **5d**, *versus* the methylene substituent in compound **3f**. **4e** also showed a statistically significant reduction in 5-LOX activity, however, lower than the activity presented by compound **5d**.

β-Amino alcohols such as **8b**, **8c**, **8e**, **8f**, **8i** and **9a** did not show statistically significant inhibition of 5-LOX, while **8d**, **8g** and **8h** showed statistically significant activity.

Another derivative that showed promising activity was 4-allylbenzene-1,2-diol **2**, which differs from the chemical structure of eugenol **1** by having two free hydroxyl groups.

**Figure 4.**
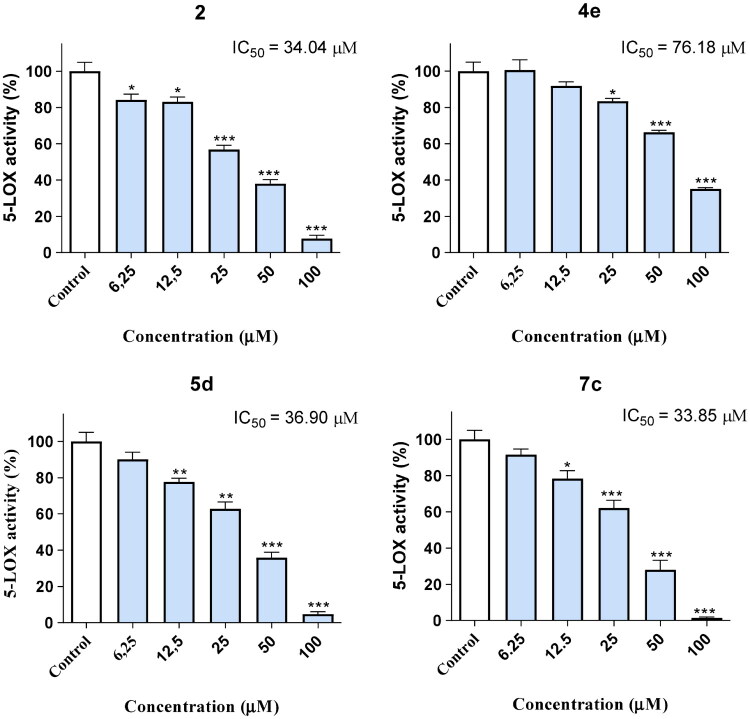
5-LOX inhibitory activity (dose-response curves) of the most active compounds. * *p* < 0.05; ** *p* < 0.01; *** *p* < 0.001.

In order to calculate the IC_50_ of the most active molecules, we selected the best-performing compounds (**2**, **4e**, **5d** and **7c**) and repeated the 5-LOX inhibitory assays, this time using a concentration range (6.25-100 µM) in order to yield dose-response data. All molecules displayed IC_50_ below 100 µM (Figure 4), with molecules **2**, **5d** and **7c** exhibiting similar IC_50_ in the 33-37 µM range.

We were then interested in further investigation the putative mechanism of action for 5-LOX inhibition. To this end, we resorted to computational studies, specifically protein-ligand docking. Initially, we compiled a list of 33 molecules described as active towards 5-LOX inhibition from CHEMBL, in addition to 8 approved drugs with the same activity, to which we added the 4 best-performing molecules from our library (Table 2). The chemical structures of all molecules selected from CHEMBL can be found in Supplementary Figure 1. Next, we calculated the docking score for all molecules using 4 different methods, namely ChemPLP score, ASP score, Chemscore and Goldscore.

**Table 2.** Docking scores obtained for all the test sets.

	Name/code	PLP score	ASP score	ChemScore score	GoldScore score
Test compounds	**2**	42.14	26.63	20.73	45.14
	**4e**	60.04	28.87	25.10	54.20
	**5d**	58.61	29.66	25.61	50.04
	**7c**	50.98	29.23	26.36	43.09
Inhibitors	Zileuton (CHEMBL93)	51.99	30.71	23.85	49.45
	Mesalamine (CHEMBL704)	45.32	24.24	21.76	43.82
	Atreleuton (CHEMBL59356)	62.13	35.68	28.04	54.62
	Meclofenamic acid (CHEMBL509)	62.23	29.50	31.89	55.98
	PF-4191834 (CHEMBL4297416)	67.15	38.68	29.36	55.83
	Olsalazine (CHEMBL425)	75.35	39.81	27.71	70.05
	Sulfasalazine (CHEMBL421)	65.46	40.31	29.41	71.15
	Balsalazide (CHEMBL1201346)	69.75	40.09	25.61	75.73
Active compounds	CHEMBL88084	75.27	38.24	33.01	57.40
	CHEMBL50170	78.56	44.77	34.48	69.28
	CHEMBL423638	69.17	40.82	32.43	68.48
	CHEMBL377821	65.97	48.14	25.07	56.41
	CHEMBL377094	65.28	46.23	23.77	59.35
	CHEMBL349526	85.36	48.30	35.81	68.58
	CHEMBL3353726	46.42	28.73	24.17	45.43
	CHEMBL32842	84.43	45.30	36.40	74.02
	CHEMBL3238465	55.97	37.17	26.07	46.36
	CHEMBL3238463	55.52	39.45	30.36	53.24
	CHEMBL3238207	56.32	37.56	23.49	50.36
	CHEMBL313489	75.84	44.59	33.13	66.72
	CHEMBL3113618	72.84	51.66	31.05	69.38
	CHEMBL3113617	65.93	37.81	28.62	66.09
	CHEMBL3113612	62.20	38.23	28.34	57.60
	CHEMBL29097	69.22	35.89	32.50	57.83
	CHEMBL280055	59.09	33.31	26.29	53.60
	CHEMBL275887	87.96	51.96	34.73	60.40
	CHEMBL275634	87.33	44.30	35.87	88.77
	CHEMBL2093045	72.72	40.37	33.26	70.71
	CHEMBL207109	64.78	41.30	28.89	58.97
	CHEMBL207057	60.92	40.33	28.48	56.87
	CHEMBL206632	68.85	44.25	26.93	57.89
	CHEMBL205800	70.71	44.21	28.58	58.77
	CHEMBL205222	71.69	44.85	32.24	73.39
	CHEMBL205189	65.55	48.19	30.51	54.75
	CHEMBL204781	64.09	43.08	33.49	53.15
	CHEMBL204633	79.16	50.26	32.67	68.95
	CHEMBL16596	91.06	42.23	35.93	58.84
	CHEMBL159516	89.63	44.89	32.55	62.88
	CHEMBL159297	84.34	40.10	34.29	62.56
	CHEMBL140915	104.88	50.95	41.94	75.90
	CHEMBL129970	60.04	30.10	27.69	65.92

**Figure 5.**
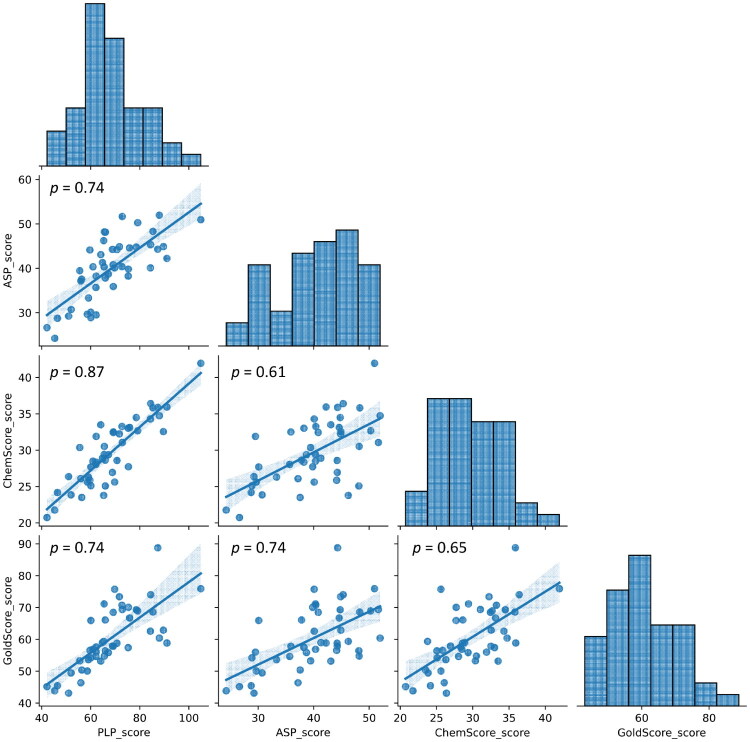
Correlation between the different docking scores used (Pearson’s correlation coefficient).

Table 2 displays the docking scores for all scoring functions and all compounds studied. GOLD scores are dimensionless and a higher score, indicates a better docking result. However, each scoring function (SF) has its strengths and limitations, depending on the type of SF that it is (Force field based, empirical or knowledge based) hence, the different scales. As shown in Figure 5, the different SF are highly correlated.

#### Correlation of docking scores to structural features of eugenol derivatives

**Figure 6.**
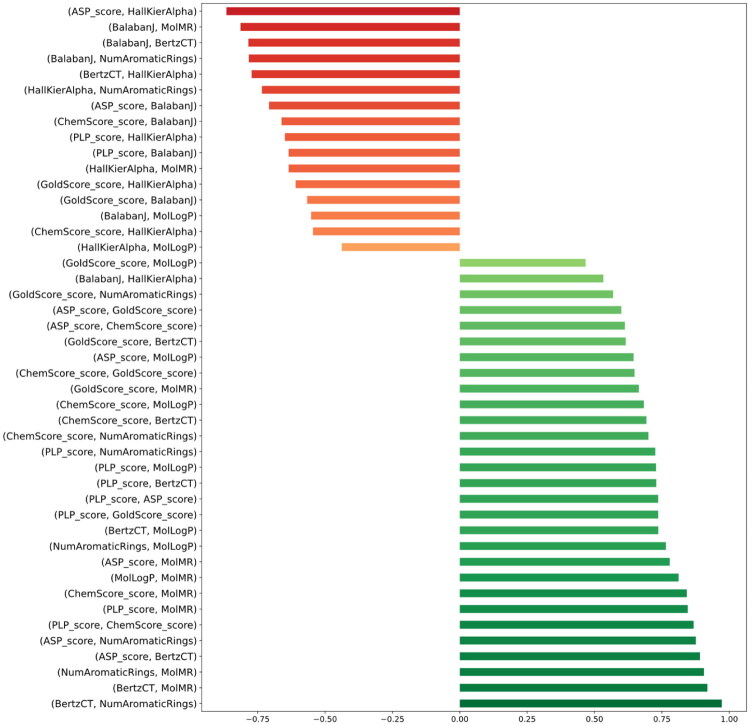
Pairwise correlation of the most correlated descriptors. **BalabanJ**: Balaban’s J value for a molecule; **BertzCT**: topological index meant to quantify complexity of molecules; **HallKierAlpha**: Hall-Kier alpha value; **MolLogP**: Wildman-Crippen LogP value; **MolMR**: Wildman-Crippen MR value; **NumAromaticRings**: number of aromatic rings.

Afterwards, we were interested in investigating if the docking scores of the molecules studied could be explained on the grounds of the physico-chemical and topographic properties of the molecules under study. In order to select the most promising molecular descriptors for this comparison, we initially used an untargeted approach in each several molecular descriptors were used in pairwise fashion to identify those most correlated to any of the docking scores employed. As showed in Supplementary Figure 2, this resulted in excessive information due to the many combinations possible. With these results, we selected only the descriptors that were exhibited the top 20 correlation coefficients (10 positive and 10 negative), namely “PLP_score”, “ASP_Score”, “Chemscore”, “GoldScore”, “BalabanJ”, “BertzCT”, “HallKierAlpha”, “NumAromaticRings”, “MolLogP” and “MolMR”. The resulting correlations are shown in Figure 6.

**Figure 7.**
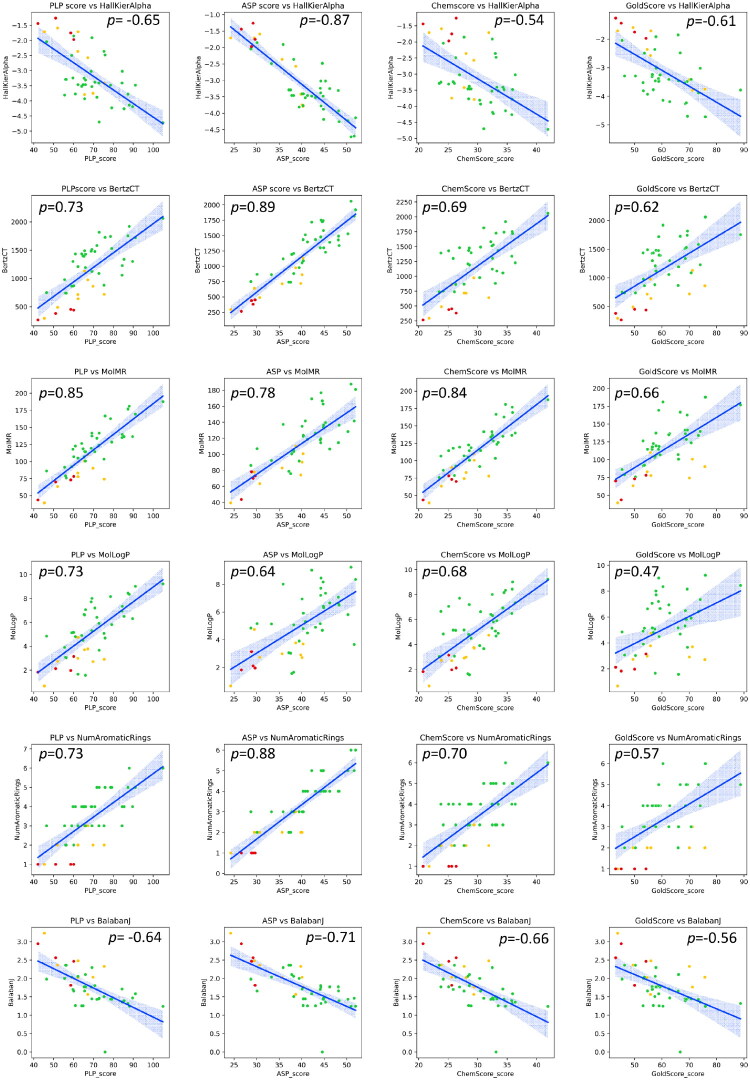
Correlation between the 4 docking scores used and the selected variables indicated, as calculated using Pearson’s correlation coefficient. Green: active molecules selected from CHEMBL, Gold: benchmark inhibitors, Red: 4 best-performing eugenol derivatives. Confidence interval: 95%. **Balabanj**: Balaban’s J value for a molecule; **BertzCT**: topological index meant to quantify complexity of molecules; **HallKierAlpha**: Hall-Kier alpha value; **MolLogP**: Wildman-Crippen LogP value; **NumAromaticRings**: Number of aromatic rings;.

From the results presented in Figure 6, we selected the variables that were more significantly correlated with any of the 4 docking scores. The results are presented in Figure 7 and point to a well-defined differentiation between the 33 best-performing molecules in CHEMBL, the 8 inhibitors selected and our 4 best-performing eugenol derivatives, in a total of 40 molecules.

**Figure 8.**
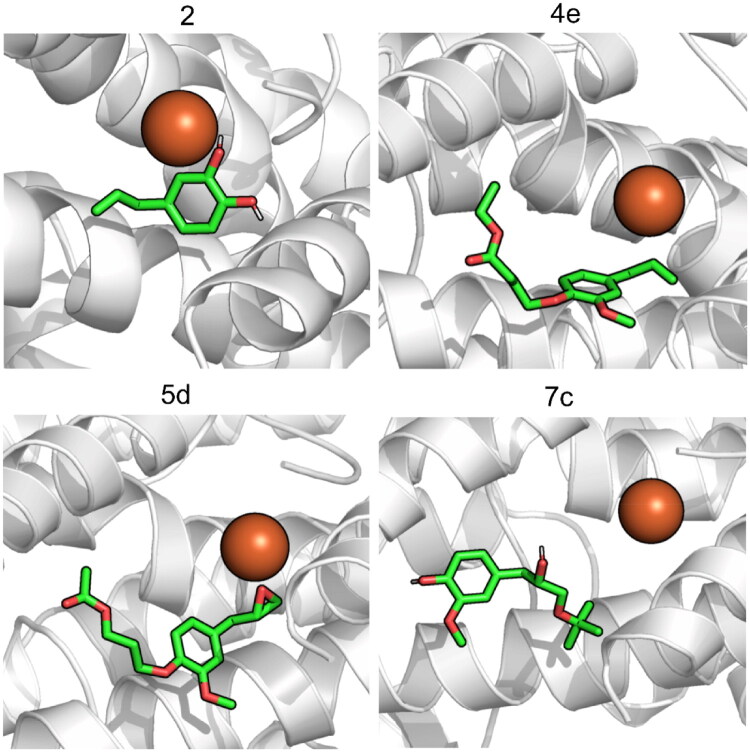
Position of compounds **2**, **4e**, **5d** and **7c** in the binding pocket of 5-LOX.

Molecular dynamics simulations were used to validate the docking predictions and the stability of the complexes. The results are shown in Table 3. All the molecules remain tightly bound to the pocket of 5-LOX with average buried areas over 95%, as seen of Figure 8. Compounds **7c** and **2** establish more hydrogen bonds with 5-LOX than compounds **5d** and **4e**, the former exhibiting H donnors, 2, as opposed to the latter (0).

**Table 3.** Average protein and ligand RMSD values (Å), Ligand RMSD (Å), Average ligand SASA (Å), Percentage of Potential Ligand SASA buried, average number of ligand-target hydrogen bonds obtained from the MD simulations. ΔG binding energy determined using MMPBSA.

	Average RMSD of the Complex (Å)	Average RMSD of the Ligand (Å)	Ligand SASA (Å^2^)	Percentage of Potential Ligand SASA buried (%)	Average Number of Hbonds	ΔGbind (kcal/mol)	Main Contributors
2	1.9 ± 0.1	0.5 ± 0.2	9.6 ± 4.2	97	1.5 ± 0.7	−12.0 ± 1.1	Ile673 (−1.9 ± 0.7) Leu368 (−1.0 ± 0.5) Leu607 (−1.0 ± 0.5)
4e	2.0 ± 0.2	1.7 ± 0.2	19.2 ± 5.2	96	0.5 ± 0.5	−13.2 ± 0.6	Tyr181 (−1.7 ± 0.8) Leu368 (−1.6 ± 0.5) Leu414 (−1.7 ± 0.5)
5d	1.9 ± 0.1	2.1 ± 0.3	10.6 ± 4.2	98	0.03 ± 0.2	−27. 2 ± 0.2	Leu368 (−2.1 ± 0.5) Ile673 (−1.9 ± 0.7) Leu414 (−1.1 ± 0.4)
7c	2.0 ± 0.1	2.0 ± 0.2	16.1 ± 5.8	97	0.9 ± 0.7	−10.8 ± 0.8	Tyr181 (−1.9 ± 0.5) Asn425 (−1.2 ± 0.9) Leu414 (−1.1 ± 0.3)

The free energy results suggest that the compounds **5d** and **4e** present the highest affinity towards 5-LOX with ΔGbind values of −27.2 and −13.2 kcal/mol, respectively. The main interacting residues seem to be Tyr181, Leu368, Leu414 and Ile673.

These results are promising in the way that they may be further used in the future for the rational design of novel drugs with improved 5-LOX inhibition. However, it must be stated that these *in silico* studies serve exploratory purposes and to not signify, at this stage, clinical potency.

### Predicted pharmacokinetics of the best-performing molecules

After the experimental and *in silico* potential of eugenol derivatives towards 5-LOX inhibition had been established, we decided to check if the putative pharmacokinetics of the molecules were favourable. We calculated several theoretical parameters predictive of pharmacokinetics, compiled in Table 4.

**Table 4.** Predicted pharmacokinetic properties for the best-performing molecules under study.

Parameter	Description	2	4e	5d	7c
**Medicinal chemistry**					
SAscore	Synthetic accessibility score ≤6: excellent	2.166	1.976	2.530	2.626
**Lipinski**		Pass	Pass	Pass	Pass
Pfizer rule	Molecules with logP > 3 and TPSA < 75 are likely to be toxic	Pass	Fail	Pass	Pass
GSK rule	Molecules with MW ≤ 400 and logP ≤ 4 have a favourable ADMET profile	Pass	Pass	Pass	Pass
PAINS	Pan Assay Interference Compounds	1	0	0	0
**Absorption**					
Caco-2 permeability	Predicted Caco-2 permeability (log cm/s) >-5.15: excellent	−4-45	−4.461	−4.583	−4.324
HIA	Human intestinal absorption 0-0.3: excellent	0.008	0.003	0.002	0.004
MDCK permeability	Predicted permeability in Madin-Darby Canine Kidney cells (cm/s) >2 × 10-6: excellent	2.60 × 10^−5^	2.85 × 10^−5^	3.50 × 10^−5^	2.08 × 10^−5^
Pgp-substrate	0-0.3: excellent (non-substrate)	0	0	0.001	0.096
F30%	Probability of bioavailability being ≥30% 0-0.3: excellent	0.983	0.56	0.058	0.013
**Distribution**					
PPB	Plasma protein binding. ≤90%: excellent	91.72	94.18	40.65	73.47
VD	Predicted volume distribution 0.04-20: excellent				
**BBB penetration**		0.053	0.472	0.568	0.428
Excretion					
CL	Drug clearance (mL/min/kg) ≥5: excellent	18.398	12.226	8.698	12.216

In addition to features related to medicinal chemistry, the parameters calculated could be grouped into the absorption, distribution, metabolisation and excretion (ADME) dimensions of pharmacokinetics. The synthetic accessibility score^38^ of all compounds shows easily assessable molecules, an experience we confirmed during synthetic steps. The molecules also passed most “rule of thumbs” used in the field, not only Lipinski’s, but also those in use by several pharmaceutical industries^39^^,^^40^. In the case of absorption, Caco-2 permeability, human intestinal absorption and MDCK permeability point to excellent expected absorption. In addition, all molecules are expected to not be clients of P-glycoprotein (P-gp), thus adding to their favourable pharmacokinetic profile. In the case of distribution, two of the molecules present high probability of showing a favourable plasma protein binding, with the remaining two molecules being very close to this trait. All molecules were also predicted to have favourable drug clearance based on their chemical structure.

## Discussion

In this work, a set of 40 molecules are assessed for their potential to be used as 5-LOX inhibitors using a suite of methodologies. Initial chemometric studies assessed the degree of conservation of the eugenol molecule in the derivatives by the means of the Tanimoto coefficient (T), which results are presented in Figure 1.

Molecule **2**, a catechol, is the molecule closer to eugenol (T = 0.61), a methoxyphenol. Molecule **4c**, which differs from **2** in the substituents of hydroxyl groups is, understandably, very close to the former (T = 0.59). The position of **3c** as the molecule with the lowest resemblance to **1** is to be expected if we consider that its pyrazine heterocycle is unique within the library and results in significantly distinct structural and topographical properties. In a general way, molecules in group **4** were closer to eugenol and group **5** was more distinct.

When several physico-chemical and topographic properties of the library were calculated and computed pairwise, results showed that for several pairs of properties, there are clear differences between molecules classified as benzenoids (**1**, **2**, **4a**-**9b**), phenylpronaoids and polyketides-like (**3d**-**h**) and organoheterocyclic compounds (**3a**,**b**), their frequency distribution having been presented in the Results section.

After this preliminary chemometric analysis, molecules were evaluated for their ability to inhibit 5-LOX. When considering the inhibitory effect of the 40 molecules under study, comparision of the structure of **7a** and **7f**, which were inactive, towards **7c**, which displayed full inhibition, the only structural difference in compound **7c** is the presence of a branched alkyl chain, particularly *tert*-butyl *versus* the phenyl substituent found in compound **7f** and the methyl substituent found in compound **7a**. Of note, **7c** is the molecule displaying the highest fraction of sp^3^ carbons, which has been identified before as a very relevant trait for enhanced biological activity^41^. When comparing **7b** with **7a**, the former exhibits around 30% of inhibition while the latter is inactive, the only difference among the two being the size of the chain by one carbon.

When comparing **5d** with **3f**, which was inactive, the only difference in compound **5d** is the presence of an oxyran substituent group *versus* the methylene substituent in compound **3f**. Another compound structurally similar to **5d** is **4e**, which also showed a statistically significant reduction in 5-LOX activity, however, lower than the activity presented by compound **5d**. As found with **3f**, the difference in inhibition potential can be explained by the presence of the allyl group in **4e** *versus* the oxyrane functional group of **5d**.

It is important to note that β-amino alcohol compounds such as **8b**, **8c**, **8e**, **8f**, **8i** and **9a** were found to be inactive, while **8d**, **8g** and **8h** showed statistically significant activity, albeit lower than the activity displayed by eugenol **1**.

When we compare the structure of the more active compound **7c** with compound **9a**, the only structural difference is the presence of the ether group in compound **7c** *versus* the amino group in compound **9a**. Therefore, it is suggested that compounds derived from β-alkoxy alcohols potentiate the inhibitory activity against LOX when compared with β-amino alcohols derivatives.

Compound **2**, which bears two free hydroxyl groups, exhibited promising activity. Data in literature^42^ show that the aromatic ring and the hydroxyl group are essential structures in a molecule to confer inhibitory activity against LOX.

The compound 2-methoxy-4-(oxiran-2-ylmethyl)phenol **6**, when compared to compound **2**, did not show inhibitory activity even though it is a short-chain molecule and this may be related to the difference in its substituent groups, since that compound **6** has a methoxy group and oxyrane *versus* hydroxyl group and open allyl chain of compound **2**. Thus, it is worth noting that even being a low molecular weight compound, the presence of open lateral structures is necessary for the maintenance of the activity.

After testing all molecules, the best-performing compounds were selected on the basis of their lower IC_50_, namely **2**, **4e**, **5d** and **7c**, all molecules displaying IC_50_ lower than 100 µM (Figure 4).

Having compiled the structure of 33 molecules described as 5-LOX inhibitors and also 8 approved 5-LOX drugs with the same target from the database CHEMBL, we added our molecules under study to these inhibitors and calculated their docking scores, using four different methods (Table 2), ultimately showing the high correlation of the different scoring functions (Figure 5). Importantly, high correlation were found between the docking scores used and the physico-chemical/topological features depicted in Figures 6 and 7. This is an important result, as it adds to the body of information that allows the selection of the molecules with the best chance of rendering potent inhibitors in future studies.

BertzCT is a topological index meant to quantify “complexity” of molecules and consists of a sum of two terms, one representing the complexity of the bonding, the other representing the complexity of the distribution of heteroatoms. As shown in Figure 7, the 4 eugenol derivatives are among the least complex molecules in the entire dataset. This is further confirmed by the fact that the 4 eugenol derivatives are the only molecules in the dataset that bear a single aromatic ring, apart from one of the benchmark inhibitors, mesalamine. This constitutes an advantage for potential use in the pharmaceutical industry, as it means that their synthesis is easier, cheaper, and less time-consuming.

In the case of logP, it is shown that around 50% of the library presents logP > 5, which is a violation to Lipinski’s Rule of 5. As expected, all the benchmark inhibitors display logP < 5, a desirable trait shared by all 4 eugenol derivatives.

Because the docking scores of the test compounds (**2**, **4e**, **5d** and **7c**) are close to the ones obtained for the inhibitor and active test sets (with r equal or superior to 0.7 - Pearson correlation), across all the scoring functions, we can conclude that they do have potential to become 5-LOX inhibitors.

Molecular dynamics simulations were conducted to validate the docking predictions and the stability of the complexes was evaluated through RMSD calculations for the Cα atoms of each complex and ligand, calculation of Solvent Accessible Surface Area (SASA) and Percentage of Potential Ligand SASA buried and hydrogen bonding analysis. The results are shown in Table 3.

The protein-ligand complexes are stable throughout the simulations, with a RMSD of around 2 Å, indicating that the binding conformation predicted in the docking stage was maintained. All the molecules remain tightly bound to the pocket of 5-LOX with average buried areas over 95%, as seen of Figure 8. Hydrogen bonding analysis is important to understand the stability of the interactions between the targets and ligands throughout time. Compounds **7c** and **2** establish more hydrogen bonds with 5-LOX than compounds **5d** and **4e**, the former exhibiting H donnors, 2, as opposed to the latter (0).

The free energy results suggest that the compounds **5d** and **4e** present the highest affinity towards 5-LOX with ΔGbind values of −27.2 and −13.2 kcal/mol, respectively. This is coherent with the results shown previously, with these compounds showing similar scores and total surface area values to the already used inhibitors. The main interacting residues seem to be Tyr181, Leu368, Leu414 and Ile673.

Subsequently, some pharmacokinetic parameters of the best-performing molecules were collected using *in silico* tools. Pharmacokinetic studies require comprehensive and complex studies, most of which relying *in vivo* experimentation. However, for upstream stages of drug development, computational tools can predict with increasing detail and accuracy the likely pharmacokinetic properties based on known functional groups and molecular fragments.

Table 4 shows that, in a general way, all molecules exhibit favourable pharmacokinetc properties, including good synthetic accessibility score^38^, several “rules of thumb” in use at the industrial level, excellent predicted absorption (Caco-2 permeability, human intestinal absorption and MDCK permeability), no predicted transport by P-glycoprotein (P-gp).

Finally, all molecules are also predicted to have excellent drug clearance based on their chemical structure. Overall, the prospects for favourable pharmacokinetics are highly encouraging.

## Conclusion

A series of semi-synthetic derivatives of eugenol **1** were prepared and initially evaluated through a preliminary screening to select the most active compounds. The results showed that some derivatives possess enhanced anti-inflammatory properties in relation to eugenol. The most promising compounds, 4-allylbenzene-1,2-diol **2**, ethyl- 4-(4-allyl-2-methoxyphenoxy)butanoate **4e**, 3–(2-methoxy-4-(oxiran-2-ylmethyl)phenoxy)propyl acetate **5d**, and 4–(3-(*tert*-butoxy)-2-hydroxypropyl)-2-methoxyphenol **7c** were selected for further analysis in order to obtain dose-response curves.

*In silico* studies suggest that the compounds **2**, **4e**, **5d** and **7c** have high affinity towards 5-LOX, presenting docking scores similar to the ones calculated for the known inhibitors, and that their positions remain very stable throughout the simulation time. We have also been able to pinpoint the structural features and physico-chemical properties most correlated to the LOX-inhibitory effect found, thus facilitating future design of increasingly potent molecules.

These findings point to the potential use of these semi-synthetic eugenol derivatives as anti-inflammatory agents. With this preliminary data, more detailed and informative experiments can subsequently take place, including *in vivo* assessment.

## Supplementary Material

Supplementary Figures.docx

## Data Availability

All data is available upon reasonable request.
