# Supplementary material for: Development of eugenol derivatives with 5-LOX inhibitory activity
Source: J Enzyme Inhib Med Chem. 2025 Aug 21;40(1):2535586. doi: 10.1080/14756366.2025.2535586 (PMC12377105; doi:10.1080/14756366.2025.2535586)

**Supplementary Figure 1** - Chemical structures of all molecules extracted from CHEMBLE and selected for this work.

**Supplementary Figure 2 -** Correlation values for several molecular descriptors calculated using RdKit, in addition to the features PLP_score, ASP_score, ChemScore and GoldScore.

**Supplementary Figure 1**


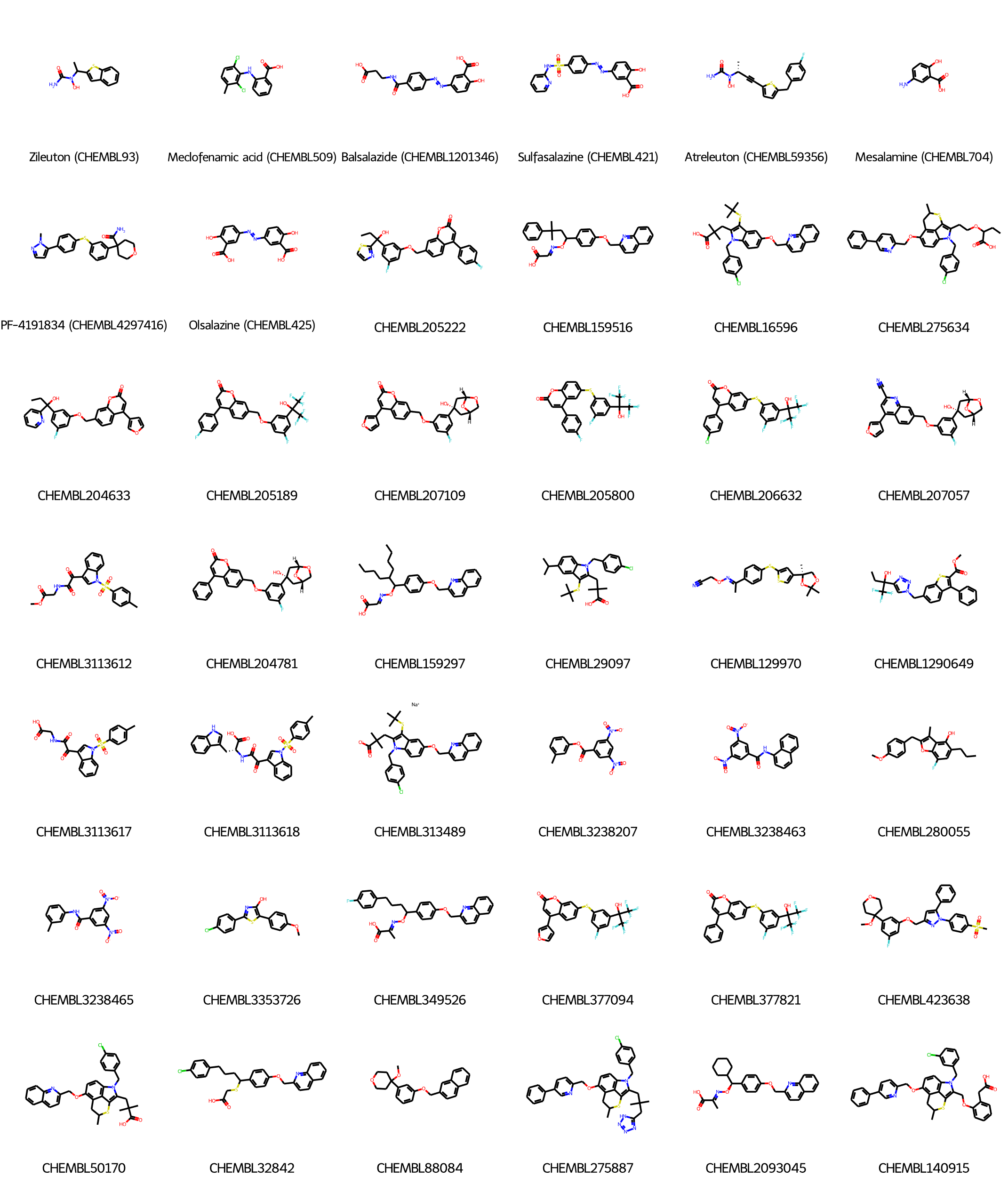


**Supplementary Figure 2**


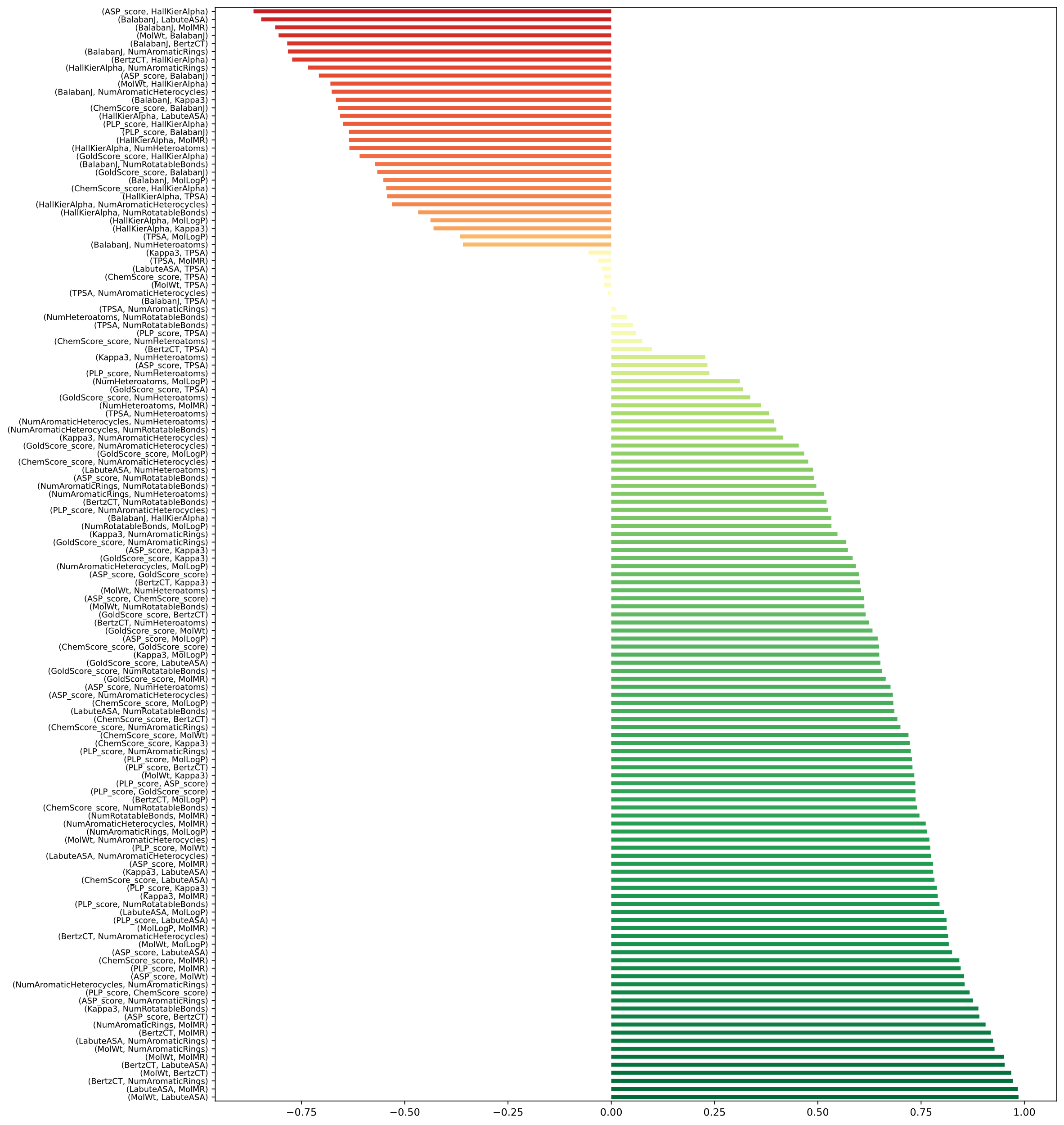

Supplement: Supplementary Figures.docx [file IENZ_A_2535586_SM7418.docx]
